# Unique features of myogenesis in Egyptian cobra (*Naja haje*) (Squamata: Serpentes: Elapidae)

**DOI:** 10.1007/s00709-015-0840-3

**Published:** 2015-05-30

**Authors:** Eraqi R. Khannoon, Weronika Rupik, Damian Lewandowski, Magda Dubińska–Magiera, Elwira Swadźba, Małgorzata Daczewska

**Affiliations:** Zoology Department, Faculty of Science, Fayoum University, Fayoum, 63514 Egypt; Department of Animal Histology and Embryology, University of Silesia, 9 Bankowa Str., 40-007 Katowice, Poland; Department of Animal Developmental Biology, Institute of Experimental Biology, University of Wrocław, 21 Sienkiewicza Str., 53-335 Wrocław, Poland

**Keywords:** Myogenesis, Satellite cells, TEM, *Naja haje*, Snake

## Abstract

During early stages of myotomal myogenesis, the myotome of Egyptian cobra (*Naja haje*) is composed of homogenous populations of mononucleated primary myotubes. At later developmental phase, primary myotubes are accompanied by closely adhering mononucleated cells. Based on localization and morphology, we assume that mononucleated cells share features with satellite cells involved in muscle growth. An indirect morphological evidence of the fusion of mononucleated cells with myotubes is the presence of numerous vesicles in the subsarcolemmal region of myotubes adjacent to mononucleated cell. As differentiation proceeded, secondary muscle fibres appeared with considerably smaller diameter as compared to primary muscle fibre. Studies on *N. haje* myotomal myogenesis revealed some unique features of muscle differentiation. TEM analysis showed in the *N. haje* myotomes two classes of muscle fibres. The first class was characterized by typical for fast muscle fibres regular distribution of myofibrils which fill the whole volume of muscle fibre sarcoplasm. White muscle fibres in studied species were a prominent group of muscles in the myotome. The second class showed tightly paced myofibrils surrounding the centrally located nucleus accompanied by numerous vesicles of different diameter. The sarcoplasm of these cells was characterized by numerous lipid droplets. Based on morphological features, we believe that muscle capable of lipid storage belong to slow muscle fibres and the presence of lipid droplets in the sarcoplasm of these muscles during myogenesis might be a crucial adaptive mechanisms for subsequent hibernation in adults. This phenomenon was, for the first time, described in studies on *N. haje* myogenesis.

## Introduction

Differentiation of the trunk (myotomal) muscles has been investigated in detail in different groups of vertebrates including fishes, amphibians, birds and mammals. The myotomal muscles in all studied vertebrate specimens originate from unsegmented paraxial mesoderm which, during subsequent stages of embryonal development, is subdivided into repetitive cell units called somites. The somites then differentiate into three compartments: the dermomyotome with a well-developed dorsomedial lip and ventrolateral lip, the sclerotome and afterwards into the myotome. The main part of the myotome is occupied by trunk muscles which differentiate in situ (reviewed by Bentzinger et al. 2012). Studies on development of amniote (birds and mammals) trunk muscles have revealed that the dermomyotome is the main source of trunk muscle progenitor cells, which express the paired-box transcription factors Pax3 and Pax7. It has been shown that Pax3 is required to establish the myogenic potential of differentiating cells, whereas Pax7 is required for the specification and maintenance of satellite cells during myogenesis (Horst et al. 2006; Buckingham and Realaix 2007; Kacperczyk et al. 2009; Seale et al. 2000; Olguin and Olwin 2004; Zammit et al. 2006).

During the vertebrates’ development, myogenic progenitors differentiate into myoblasts, which differentiate into myotubes, a developing immature skeletal muscle fibres with a centrally located nucleus in the sarcoplasm. The final stage of myogenesis is a formation of mature muscle fibres—cylindrical multinucleate cells with peripherally located nuclei. Muscles grow through two mechanisms: hypertrophy and hyperplasia. During hypertrophy, the fibre size increases due to the addition of new nuclei into sarcoplasm of pre-existing fibres, while hyperplasia is a process of new muscle fibre formation. It is well evidenced that in amniotes, Pax7 muscle progenitor cells participate in both processes in the prenatal stage. It is noteworthy that, after birth, a number of muscle fibres remain unchanged in mammals and birds (Rowe and Goldspink 1969; Fowler et al. 1980; Nimmo and Snow 1983; Brown 1987; Rosenblatt and Woods 1992; Schadereit et al. 1995). Postnatal growth is mainly due to hypertrophy, although some reports have revealed an increase in fibre number shortly after birth (Swatland 1975; Rehfeldt and Fiedler 1984; Summers and Medrano 1994; Fiedler et al. 1998).

Among all vertebrates, reptile myogenesis and myotomal muscle growth are poorly known. Studies on reptile myogenesis have been conducted on several species (Chinese soft-shelled turtle *Pelodiscus sinensis* and sand lizard *Lacerta agilis*); among them, only sand lizard studies on myogenesis shed light on the origin of muscle progenitor cells and growth of myotomal muscles (Nagashima et al. 2005; Rupik et al. 2012). It was found that in the sand lizard, similarly to birds and mammals, the dermomyotome is the main source of muscle Pax3-positive progenitor cells. Studies conducted by Rupik et al. (2012) revealed that muscle growth in the sand lizard is due to Pax3/Pax7-positive mononucleated cells. In comparison to the sand lizard, snakes, e.g. *Naja haje*, represent a different mode of locomotion. In our studies, we attempted to answer the question whether mode of locomotion influences the pattern of muscle differentiation and growth. In the present study, we investigated trunk muscle differentiation and growth in the previously unstudied Egyptian cobra (*N. haje*) in order to demonstrate whether myogenesis in the studied species displays a unique character, not observed in other vertebrates.

## Material and methods

The Egyptian cobra (*N. haje* L. (1758): Elapidae, Reptilia) is one of the largest cobra species native to Africa. It is terrestrial and crepuscular or nocturnal. Adult female cobras attain sexual maturity after 2 to 3 years. They lay 17 to 22 eggs in a clutch. Oviposition occurs 60–100 days after copulation in early summer (Schleich et al. 1996). Fertilized female Egyptian cobras for this study were collected from the Nile Delta region of Egypt in June 2012–2013. The animals were kept in vivaria in an open farm area, in conditions similar to those in the wild, until the eggs were laid, and then they were released into their native area. All specimens used in the experiment were captured according to the Egyptian regulations concerning the protection of wild species (Convention on Biological Diversity ratified in 1992 and 1994). The Egyptian cobra is not included in the Washington Convention of 1973. The eggs of Egyptian cobra (*n* = 100) after oviposition were carefully collected and brought to the laboratory of the Zoology Department, Faculty of Science, Fayoum University, where they were placed in plastic boxes filled with moistened perlite (at 85–90 % moisture) and with ventilation holes. The eggs were incubated at 30 °C, reflecting ambient seasonal temperatures in the wild. Embryos used for investigation were isolated at regular intervals from egg laying to hatching. Embryonic development at 30 °C in laboratory conditions from when the eggs are laid until they hatch lasts 51–54 days (Khannoon and Evans 2014). The age of the embryos was calculated using the table for *N. haje* development (Khannoon and Evans 2014). The study of myotomal muscle differentiation and growth of *N. haje* included four developmental stages from stages 3 to 6.

For light and electron microscopic techniques, small pieces of embryonic body wall including differentiating muscle tissue were fixed in a 1:1 mixture of 2.5 % glutaraldehyde (Sigma-Aldrich) and 2.0 % paraformaldehyde (Sigma-Aldrich, St. Louis, MO, USA) in 0.1 m phosphate buffer, pH 7.4, for 24 h at 4 °C. The material was repeatedly rinsed in the same buffer and post-fixed for 2 h in 1 % OsO_4_ (Sigma-Aldrich) in 0.1 M phosphate buffer. Following rinsing in phosphate buffer, the material was dehydrated first in a graded alcohol series and then in acetone and embedded in epoxy resin Epon 812 (Sigma-Aldrich) (Luft 1961). This procedure of fixation appears to be the best for different embryonic reptilian tissues (Rupik 2002, 2011, 2012, 2013; Swadźba and Rupik 2010, 2012). The Epon blocks were cut on Leica Ultracut UCT (Leica, Wetzlar, Germany) and Reichert Ultracut E ultramicrotome (Leica, Wetzlar, Germany). Semi-thin sections (0.6 μm) were collected on glass slides and stained with methylene blue in 1 % borax solution (Sigma-Aldrich) and examined under a light microscope Olympus BX60 and Olympus BHS light microscopes (Olympus Corp., Tokyo, Japan). Ultrathin sections were collected on 200 mesh copper grids, stained with uranyl acetate and lead citrate according to the standard protocol (Reynolds 1963) and examined in a Hitachi H500 (Hitachi Ltd., Tokyo, Japan; 75 kV) and Zeiss EM 900 (Carl Zeiss AG, Oberkochen, Germany; 80 kV) transmission electron microscopes. Histological material was processed and analysed in the Department of Animal Histology and Embryology, University of Silesia, and in the Department of Animal Developmental Biology, Institute of Experimental Biology, University of Wrocław.

## Results

### Myotomal muscles differentiation and growth in *N. haje*

**Fig. 1:**
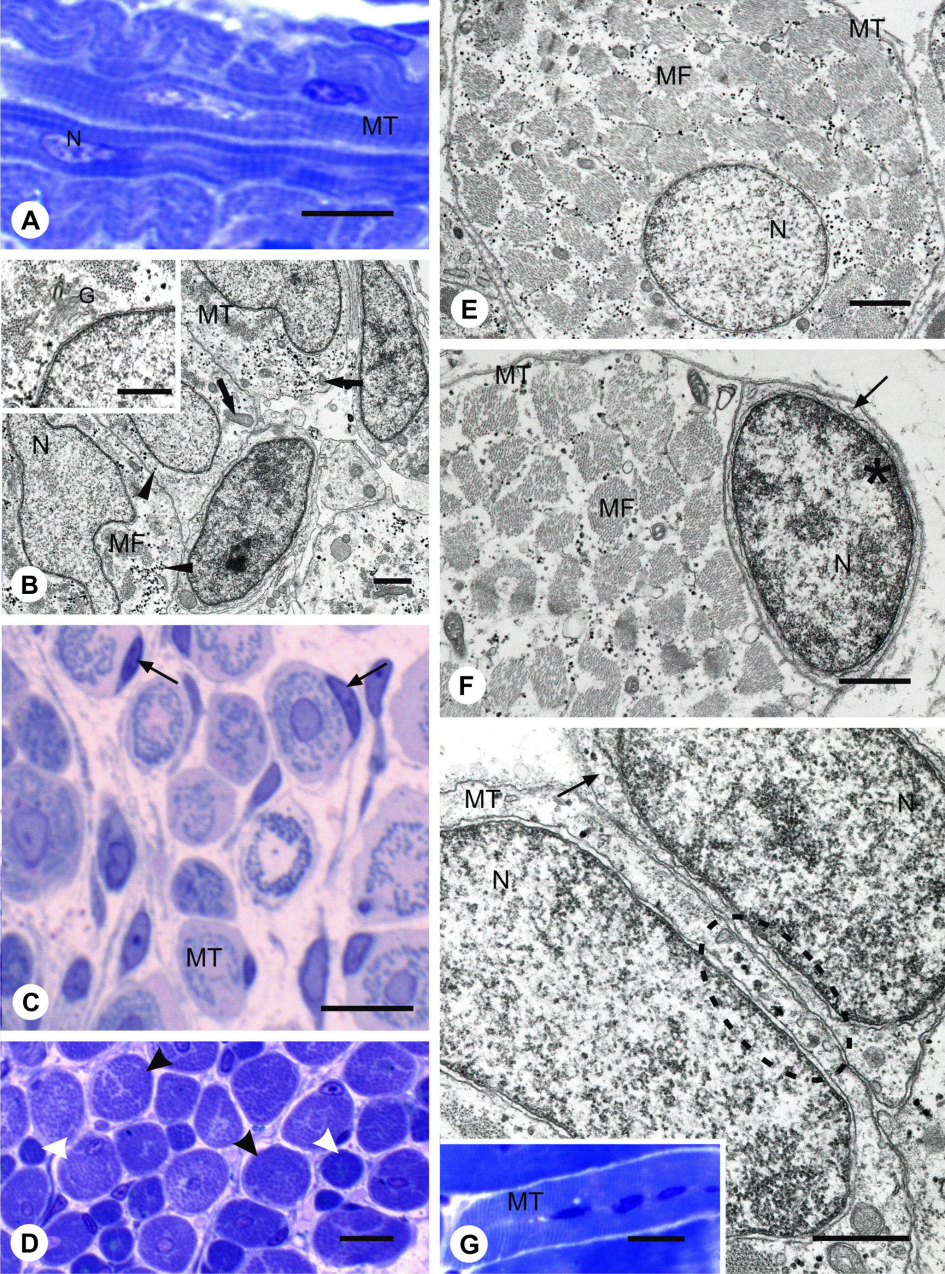
Myotomal muscles differentiation and growth in *N. haje*. **a** Stage 3: Longitudinal section of the posterior trunk myotome. Mononucleated myotubes (*MT*) centrally located homogenous nucleus (*N*). Semi-thin section, methylene blue staining, *scale bar*: 10 μm. **b** Stage 3: Ultrastructure of the posterior trunk myotome. Myotubes (*MT*), myofibril (*MF*), nucleus (*N*), glycogen (*black arrowhead*), mitochondria (*black arrows*). *Inset*: Golgi apparatus (*G*). TEM, *scale bar*: 1 μm. **c** Stage 4: Cross-sections of the posterior trunk myotome. Primary myotube (*MT*), mononucleated cells (*black arrows*). Semi-thin section, methylene blue staining, scale bar: 10 μm. **d** Stage 5: Cross-sections through the posterior trunk myotome. Primary myotubes (*black arrowheads*), secondary myotubes (*white arrowheads*). Semi-thin section, methylene blue staining, *scale bar*: 10 μm. **e** Stage 5: Cross-sections through the posterior trunk myotome. Ultrastructure of myotubes (*MT*), myofibrils (*MF*), nucleus (*N*). TEM, *scale bar*: 1 μm. **f** Stage 5: Cross-sections through the posterior trunk myotome. Myotube (*MT*), myofibril (*MF*), mononucleated cell (*black arrow*), nucleus (*N*), heterochromatin (*asterisk*) under the nuclear envelope. TEM, scale bar: 1 μm. **g** Stage 5: Cross-sections through the posterior trunk myotome. Mononucleated cell (*black arrow*), myotube (*MT*), vesicles in the subsarcolemmal sarcoplasm of the myotubes plasma membranes (*circled*), myotube nucleus (*N*) nucleus of mononucleated cell (*N*). *Inset*: multinucleated myotube (*MT*). TEM, *scale bar*: 1 μm

Myotomal muscle formation of *N. haje* was analysed in the light microscope and TEM. At stage 3, the myotome of *N. haje* is composed of homogeneous populations of mononucleated primary myotubes containing centrally located homogeneous nuclei (Fig. 1a). Sarcoplasm of these cells revealed the presence of a few myofibrils arranged in an irregular way, numerous mitochondria, Golgi apparatus and glycogen granules. At this stage of development, neighbouring myotubes are closely attached to each other (Fig. 1b). At later developmental phase (stage 4), light microscope analysis revealed that primary myotubes are accompanied by closely adhering mononucleated cell (Fig. 1c). As differentiation proceeded (stage 5), secondary muscle fibres appear. The secondary muscle fibres are distinguished by considerably smaller diameter as compared to primary muscle fibre (Fig. 1d). At this stage of myogenesis, numbers of myofibrils in sarcoplasm of myotubes were higher (Fig. 1e). Ultrastructural analysis showed that mononucleated cells closely adhering to myotubes are composed of a prominent nucleus with heterochromatin located mainly under the nuclear envelope. The nucleus is surrounded by a narrow rim of cytoplasm devoid of glycogen granules and elements of contractile apparatus (Fig. 1f). Localization of mononucleated cells suggests their potential involvement in muscle growth due to their fusion with myotubes. This is plausible since the detailed ultrastructural analysis showed the presence of numerous vesicles in the subsarcolemmal region of myotubes adjacent to mononucleated cells. At this developmental stage, as a result of progressing myogenic cell fusion, elongated, multinucleated myotubes appear (Fig. 1g).

### Unique features of muscle differentiation in *N. haje*

**Fig. 2:**
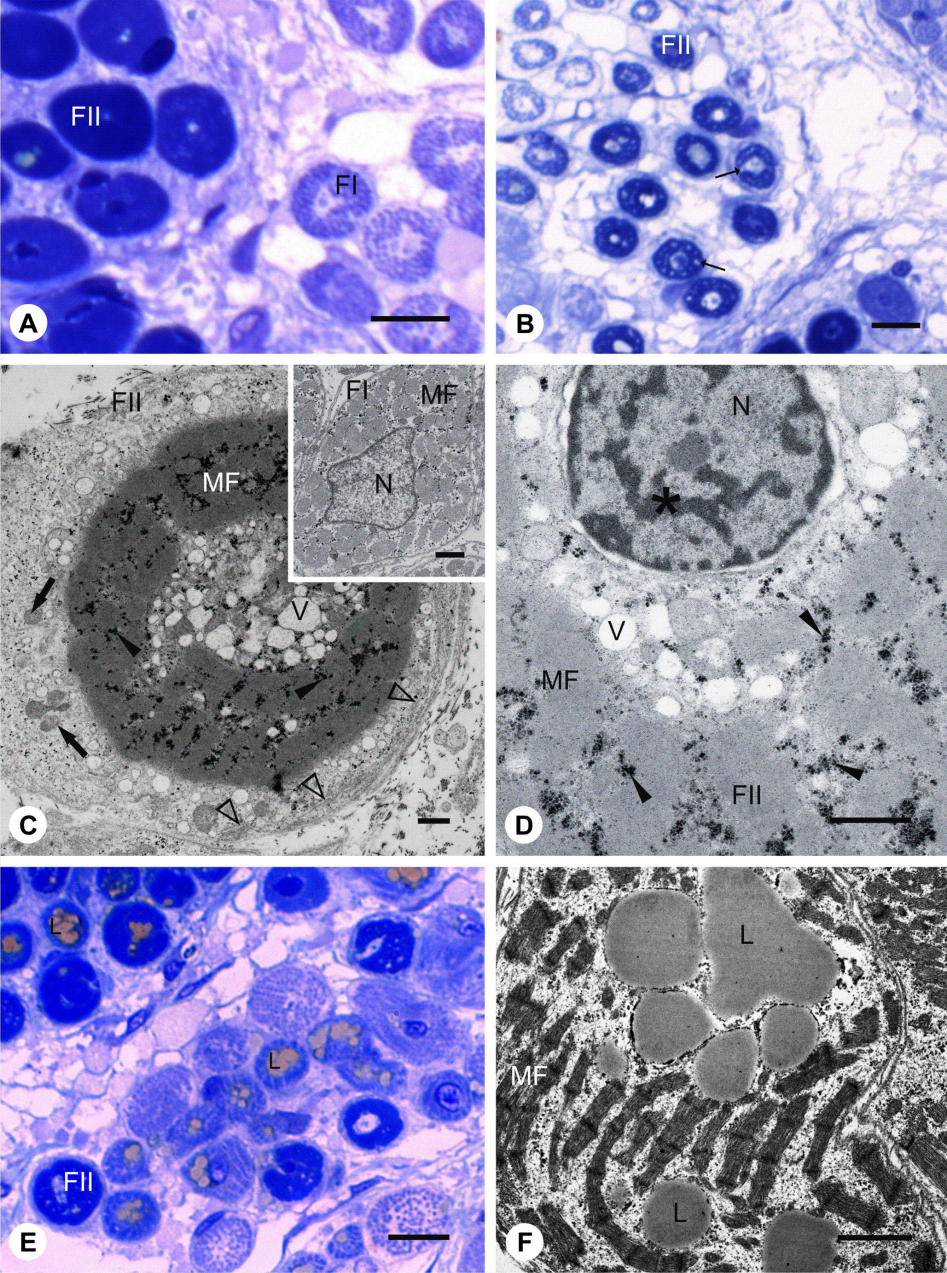
Unique features of muscle differentiation in *N. haje*. **a** Stage 5: Cross-sections through the posterior trunk myotome. Heterogeneous population of muscle fibres in the myotome. First class of muscle fibres (*FI*), second class of muscle fibres (*FII*). Semi-thin section, methylene blue staining, *scale bar*: 10 μm. **b** Stage 5: Cross-sections through the posterior trunk myotome. Class II of muscle fibres (*FII*), vesicles (*black arrows*). Semi-thin section, methylene blue staining, *scale bar*: 10 μm. **c** Stage 5: Cross-sections through the posterior trunk myotome. Ultrastructure of class II of muscle fibres (*FII*). Myofibrils (*MF*), vesicles (*V*), glycogen granules (*black arrowheads*), mitochondria (*black arrows*), endoplasmic reticulum (*empty arrowheads*). *Inset*: Ultrastructure of class I muscle fibres (*FI*). Myofibrils (*MF*), nucleus (*N*). TEM, *scale bar*: 1 μm. **d** Stage 5: Cross-sections through the posterior trunk myotome. Ultrastructure of class II of muscle fibres (*FII*). Myofibrils (*MF*) nucleus (*N*) rich in heterochromatin (*asterisk*), vesicles (*V*), glycogen granules (*black arrowheads*). TEM, *scale bar*: 1 μm. **e** Stage 6: Cross-sections through the posterior trunk myotome. Numerous lipid droplets (*L*) in sarcoplasm of class II of muscle fibres (*FII*). Semi-thin section, methylene blue staining, *scale bar*: 10 μm. **f** Stage 6: Longitudinal through the posterior trunk myotome. Ultrastructure of class II of muscle fibres. Numerous lipid droplets (*L*) in the sarcoplasm, myofibrils (*MF*). TEM, *scale bar*: 1 μm

During myotomal myogenesis (stage 5), developing muscle fibres of *N. haje* do not form a homogeneous population. Undertaken studies revealed the presence of two classes of muscle fibres distinguished by the distribution of myofibrils. Class I is characterized by typical regular distribution of myofibrils which fill the whole volume of the muscle fibre sarcoplasm (Fig. 2a). Class II features tightly packed myofibrils surrounding a centrally located nucleus accompanied by numerous vesicles. They are localized only in the proximity of the nucleus and do not occur in the peripheral region of the sarcoplasm, which is filled with numerous mitochondria and endoplasmic reticulum. Sarcoplasm of II class of muscle fibres contains numerous glycogen granules among myofibrils and in the peripheral region (Fig. 2b, c). The nuclei of class II muscle fibres are rich in heterochromatin localized beneath the nuclear envelope and in the internal part of the nucleus (Fig. 2d). Sarcoplasm of these cells is also characterized by numerous lipid droplets surrounded by glycogen granules (Fig. 2e, f).

## Discussion

### Differentiation and growth of muscles in *N. haje*

Skeletal muscle differentiation and growth in vertebrates have been investigated in detail in model species (zebrafish, *Xenopus laevis*, chick, mouse) (reviewed by Bentzinger et al. 2012; Kiełbówna and Jędrzejowska 2012; Rossi and Messina 2014). These model vertebrate species provided detailed information at the morphological and the molecular level of muscle development in this phylum. Studies on reptilian developmental biology including myogenesis are difficult and rarely attempted (Nagashima et al. 2005; Rupik et al. 2012; Kusumi et al. 2013). To better understand the mechanisms of trunk muscle differentiation and growth in reptiles, we chose the Egyptian cobra. Our results revealed that in the studied species during early steps of myogenesis, the myotome was composed of a homogeneous population of myogenic cells consisting of mononucleated primary myotubes. The structure and morphology of these cells in *N. haje* myotomes resemble mononucleated myocytes observed in primary myotomes in birds and mammals and also in the sand lizard, a representative of reptilians which, in comparison to Egyptian cobra, represents a different mode of locomotion. During subsequent stages of muscle fibres differentiation, myotubes were accompanied by mononucleated cells. Based on localization and morphology, these cells share features with satellite cells, described in detail in many vertebrates (fish, amphibians, birds and mammals) (Zammit et al. 2006; Kacperczyk et al. 2009, 2011; Daughters et al. 2011; reviewed by Siegiel et al. 2013; Yin et al. 2013). TEM analysis revealed that satellite cells in the studied species are spindle-shaped composed of a prominent nucleus with heterochromatin located beneath the nuclear envelope. The narrow rim of their cytoplasm is devoid of glycogen granules and elements of contractile apparatus. Compared to differentiating myotubes, the ultrastructure of satellite cells showed an undifferentiated state. In the sand lizard, similarly to birds and mammals, satellite cells that adjoin the differentiating muscle fibres express Pax7 protein (Rupik et al. 2012). The origin of these cells in *N. haje* has not been studied. Many different lines of evidence indicate that in amniotes, mononucleated cells that adjoin the myotubes and/or muscle fibres originate from the dermomyotome. Studies on sand lizard myogenesis revealed that, as in other vertebrates, these cells are myogenic precursors involved in muscle growth (both hypertrophic and hyperplastic) (Rupik et al. 2012). Increase in the number of nuclei in myotubes (hypertrophy) in subsequent stages of myogenesis and occurrence of new muscle fibres (hyperplasia) with smaller diameter compared to the primary muscle fibres is indirect evidence of participation of these cells in *N. haje* muscle growth. The presence of numerous vesicles budding from the myotube sarcolemma, closely adhering to the surface of the myotubes, is morphological evidence of the fusion of these cells in *N. haje*. Similar vesicles have also been found below the plasmalemma of fusing chicken myoblasts in vitro and in vivo during teleost muscle growth. During muscle growth in the sand lizard, detailed TEM analysis also showed numerous vesicles in the subsarcolemmal sarcoplasm of muscle fibres and the partial fusion of muscle fibre and mononucleated cell plasma membranes (Kalderon and Gilula 1979; Merkel 1995; Daczewska 2001; Kacperczyk and Daczewska 2006, 2008; Rupik et al. 2012). Data obtained from these studies strongly support the hypothesis that mononucleated cells/satellite cells in *N. haje* participate in muscle growth. Our studies revealed that the pattern of muscle differentiation and growth in reptiles is independent of the mode of locomotion (see lizards versus snakes).

Locomotor adaptations are essential for survival as locomotion plays a crucial role in many biological functions including the capturing of prey, competing with possible rivals and escaping predators (Garland and Losos 1994; Irshick and Garland 2001; Aubret 2004). Locomotion strongly differs from that of four-legged taxa whose propulsion and support are mainly ensured by the limbs (Aerts et al. 2000; McElroy and Reilly 2009). Indeed, snakes can use their entire body to generate propulsion when in contact with the substrate. The axial muscles of snakes are notable for having long tendons within individual segments that span several vertebrae. Consequently, muscles that extend anteriorly have a constraint on their length as their origins locate closer to the skull. Compared to other amniotic vertebrates, snakes have extraordinarily long axial muscles in which the contractile tissue commonly spans 3–6 vertebrae, and tendons may span from one to more than 30 vertebrae, depending on the particular muscle and species (Mosauer 1935; Gasc 1981; Jayne 1982;). In lizards, axial muscles stabilize the trunk during locomotion, and according to Ritter (1996), this stabilizing role is a basal feature of lizards. Enzymatic and histochemical analysis revealed that lizards possess both slow and fast muscles (proportion of muscle types depends on locomotor behaviour) whereas white muscle fibres in snakes represent a prominent group of muscles in myotomes (Gleeson et al. 1980; Guthe 1981; Moritz and Schilling 2013). Data obtained from studies on muscle differentiation and growth in *N. haje* provide new evidence confirming conservation of the myogenic programme in amniotes. Further research on reptile myotomal myogenesis should focus on the red and white muscle differentiation to show differences among muscle fibres precursors that will give a rise the adult muscle fibres.

### Unique features of muscle differentiation in *N. haje*

Although muscle differentiation and growth in *N. haje* share great many similarities to myogenesis described in amniotes (in birds, mammals and sand lizard), our studies revealed some unique features of muscle differentiation in the studied species. As development proceeded, light microscope and TEM analysis revealed in the *N. haje* myotomes two classes of muscle fibres. The first class was characterized by typical for white/fast muscle fibres regular distribution of myofibrils which fill the whole volume of the muscle fibre sarcoplasm. White muscle fibres in studied species are a prominent group of muscles in the myotome. It has been reported that majority of fibres in snake body musculature are fast muscle fibres that are important for quicker movements and for shortening to do work (Guthe 1981). The second class showed tightly packed myofibrils surrounding the centrally located nucleus accompanied by numerous vesicles of different diameter. We suppose class I muscle fibres originate from primary and secondary myotubes, whereas class II muscle fibres may originate only from primary myotubes. This assumption was based on morphological observation regarding diameter of myotubes and muscle fibres (Kacperczyk et al. 2011). It is commonly accepted that in vertebrates, during myogenesis, secondary muscle fibres have smaller diameter compared to the primary muscle fibres. A characteristic feature of the second class of fibres is the presence of peripheral sarcoplasm containing no contractile apparatus. The sarcoplasm of these cells is also characterized by numerous lipid droplets. This phenomenon was, for the first time, described in our studies on *N. haje* myogenesis. It is commonly accepted that reptiles, as ectothermic animals, undergo hibernation as an adaptation to different habitats. Hibernation occurs also during the *N. haje* annual cycle. El-Deib (2005) described the lipid changes in blood serum and tissues of the Egyptian cobra. Applying physiological methods, the author determined lipid levels during different phases of the hibernation cycle (fatty acids, triglycerides, phospholipids and total cholesterol) in blood serum, liver, brain and cardiac and skeletal muscles. Data obtained from the study demonstrated that accumulation of lipids in the pre-hibernation phase is significantly high. During hibernation, the value of lipids significantly decreases, which suggests the consumption of lipids. Our results showed that during myogenesis in *N. haje*, there are some muscles that are capable of storing lipid droplets as the most economical form of storing energy. These muscles are also characterized by specific morphology (tightly packed myofibrils, large rim of sarcoplasm free from contractile apparatus and lipid droplets). Based on these morphological features, we believe that muscles capable of lipid storage belong to red/slow muscle fibres. The main part of the myotome in studied species is occupied by white/fast muscle fibres. These data strongly suggested that presence of lipid droplets in the sarcoplasm of some muscles during myogenesis might be a crucial adaptive mechanism for subsequent hibernation in adults.

## Perspectives

Based on biology, reptiles are unique among vertebrate taxa. Snakes, closely related to lizards, are an extremely diverse group of reptiles. Studies on Egyptian cobra myotomal muscle differentiation and growth revealed similarities and differences compared to myogenesis described in amniotes including lizards. Based on our research, we revealed that the muscle fibre differentiation in studied species shares features with lizards, e.g. myoblasts fusion leads to multinucleated primary myotubes formation, and muscle fibre growth is accompanied by mononucleated cells whereas differences are connected with the presence of two classes of muscle fibres. In *N. haje*, the first class of muscle fibres represent typical muscle fibres morphology while the second class is characterized by sarcoplasmic lipid droplets surrounded by glycogen granules, the feature never observed during *L. agilis* myogenesis (Daczewska, unpublished data).

Our research for the first time revealed that the pattern of muscle differentiation depends on specific features of Egyptian cobra biology. The results of the present study have provided some answers to questions about the mechanisms of muscle differentiation and growth in the Egyptian cobra. However, many questions remain unanswered. Studies on muscle differentiation in reptiles are incomplete and require more detailed investigation, e.g. to find the origin of muscle progenitor cells in snakes and to define regulatory factors that control muscle fibre differentiation. The main question is: In what manner may the adaptation to different environmental conditions influence the mode of myogenesis? We believe that data obtained from Egyptian cobra myogenesis provide the basis for further reptilian investigation.
